# Studying supplier-induced demand in healthcare: an econophysics approach

**DOI:** 10.3389/frhs.2025.1704835

**Published:** 2025-12-05

**Authors:** Dimitris Zavras

**Affiliations:** Laboratory for Health Technology Assessment, Department of Public Health Policy, School of Public Health, University of West Attica, Athens, Greece

**Keywords:** supplier-induced demand, market failure, econophysics, economic forces, relativistic concept, agency relationship, asymmetric information

## Abstract

Supplier-induced demand occurs when the supplier or physician, acting on behalf of the consumer or patient, generates a level of demand that surpasses what the consumer would have chosen if fully informed. Supplier-induced demand indicates the level of demand that exists beyond what would be seen in a market where consumers have complete information. In other words, the agency relationship between a patient and a physician stems from the patient's limited understanding of health, diagnosis, and treatment, which creates information asymmetry, requiring dependence on the physician's guidance. A perfect agent would suggest therapies that match the patient's needs and preferences, taking into account aspects like health issues and financial situations. Although health economics plays a critical role in understanding supplier-induced demand, this research seeks to illustrate the econophysics viewpoint of this occurrence. It specifically aims to clarify the mechanism by which supplier-induced demand develops, employing the principles of Newtonian physics. In this context, we propose interpreting supplier-induced demand through an econophysical perspective by integrating ideas from physics and economics to develop a novel conceptual framework. The mathematical structure of econophysics allows us to reproduce results through a single equation, indicating that specific patterns require monitoring.

## 1 Introduction

Among other factors, the difference between the healthcare market and ordinary markets is attributed to inherent failures that create inefficiencies if left unregulated. Supplier-induced demand (SID) is such a failure (1). SID refers to excessive utilization in the healthcare industry that is induced by physicians (2). The National Library of Medicine characterizes overuse as the inappropriate or excessive use of healthcare services (3), whereas healthcare that is not medically required is characterized as unnecessary (4). SID corresponds to the condition under which supply and demand are met, but demand and healthcare needs are not linked (5). There is strong evidence of the existence of SID, and of the impact of various forms of financial incentives on clinical practice (6).

Based on pure health economics, the theory of SID has two core groups of fundamental assumptions. According to the first group, i.e., the demand-side assumption: (i) consumer tastes, income and prices of healthcare services are included among the determinants of the demand for healthcare; (ii) marginal utility of health ($\frac{\Delta U}{\Delta H}$), marginal effect of medical care on health ($\frac{\Delta H}{\Delta M}$), and the health status of individuals influence consumer tastes for healthcare; (iii) the demand curve for healthcare will slope downward in relation to price since increasing medical care is linked to declined marginal utility of healthcare; and (iv) changes in any of $\frac{\Delta U}{\Delta H}$, $\frac{\Delta H}{\Delta M}$, or the initial health status result in individual taste changes, and in turn, shift the demand curve. According to the second group i.e., the supply-side assumption: (i) each physician faces a demand curve that is contingent on market demand and the number of physicians available for a specific population; (ii) as the number of physicians grows in relation to the population, the demand curve for each physician shifts inward; (iii) through influencing the healthcare user's perceived values of H and $\frac{\Delta H}{\Delta M,\;}$, each physician can induce a change in the healthcare user's tastes and hence an outward shift in demand for that physician's services; (iv) there exists a boundary to how much this demand inducement can take place, primarily because the physician is not the sole provider of information, and if the patient feels that the physician's information diverges significantly from reality, the patient may seek another physician; additionally, because inducement can result in disutility for the physician. In both situations, excessive SID will negatively impact the physician; and (v) it is assumed that physicians possess upward-sloping marginal cost (MC) curves; meaning, as additional services are delivered, marginal costs rise (7).

With respect to the factors influencing SID, such demand is described as occurring when the supplier or the physician, functioning as an agent for the consumer or the patient, creates a demand level that exceeds what the consumer would have selected had they been completely informed. SID represents the extent of demand that exists beyond what would occur in a market where consumers have full information; when consumers are completely informed, the demand for healthcare decreases (8). That is, the agency relationship between a healthcare user and a physician arises from the healthcare user's lack of knowledge about health, diagnosis and treatment, i.e., due to asymmetric information, necessitating reliance on the physician's advice. An ideal “perfect” agent would recommend treatments aligned with the patient's needs and preferences while considering factors such as medical conditions and financial circumstances. However, physicians also have a financial interest in providing services, potentially biasing the information given to patients. This asymmetric information, combined with extensive insurance coverage, diminishes patients' price sensitivity. When physicians operate as “imperfect” agents, they may adjust diagnoses and treatment recommendations to increase their economic well-being, resulting in more services than necessary. The balance of SID is theorized as a trade-off between the physician's increase in income and the disutility of providing unnecessary care. While low levels of demand induction may incur minimal “psychic costs,” higher levels, particularly unnecessary treatments, generate greater disutility, aligning the “psychic cost” with the marginal utility of income. Thus, SID is determined at a point where the cost of additional care equals the value derived from increased income (9).

In summary, as noted by Zweifel et al. (10), physicians have a significant effect on patient demand because of their greater level of knowledge. SID, succeeds when physicians act according to their own interests rather than as ideal agents, especially by repeatedly changing the information provided to patients in reaction to an increase in physician numbers to safeguard their jobs. Furthermore, in a context where patients possess full insurance and fees are determined externally, rational physicians whose satisfaction is affected by earnings, hours worked, and demand stimulation are anticipated to generate demand, resulting in a positive link between physician density and the per capita amount of medical services; in the case of very low physician density, this happens due to decreased rationing, resulting in a strictly proportional relationship. Additionally, the relationship between services per capita and the fee level is ambiguous, as the substitution effect indicates an increase, while the income effect points to a reduction in service volume. Moreover, the concurrent rise in per capita utilization of medical services along with a growth in physician density does not indicate that physicians generate demand. Other possible explanations for the same occurrence include the chance that current excess demand might lessen, the total cost of patient care might have reduced, and variations in physician density could arise from shifts in the demand for healthcare services. Additionally, numerous empirical studies indicate a statistically significant correlation between the concentration of physicians and per capita expenditure on healthcare services. In several cases, though, alternate explanations of reverse causality or rationing cannot be definitively ruled out.

Despite the influence of health economics in interpreting SID, this study aims to demonstrate the econophysics perspective of this phenomenon. Specifically, it seeks to explain the process through which SID arises using the principles of Newtonian physics.

## 2 Supplier-induced demand: an econophysics approach

In demand theory, similar to Newtonian mechanics, a price change results in a change in quantity demanded. While momentum change corresponds to force in mechanics, demand theory lacks a corresponding operational definition of force. Mendel developed a dynamic theory of demand borrowing from Newton's motion laws to fill this gap in economics (11).

By applying this theory, we aimed to study supplier-induced demand in the healthcare sector. To achieve the aim of this research, connecting economics with physics necessitates that at any instant, a particle possesses a particular velocity; likewise, a consumer requires a certain amount of goods and/or services. To illustrate the economic perspective of SID in healthcare, we initially highlight the parallels between physics and economics in terms of demand, as outlined by Mendel. Within this framework, multiple analogies can be identified: (i) Position *q*: By defining the economic analog of physical space as commodity space, we designate a coordinate dimension in commodity space as an account. Classifying goods of a similar nature as fungible, which economists label commodities, enables us to summarize the total amount of a commodity passing hands, i.e., *q*, by a single account balance number; (ii) Velocity *V*: $V=\frac{\mathrm{dq}}{\mathrm{dt}}$ indicates the quantity demanded; (iii) Acceleration *α*: $\alpha =\frac{\mathrm{dV}}{\mathrm{dt}}$ reflects the needs; (iv) Mass m: m denotes the price inelasticity, with elasticity $\varepsilon =\frac{1}{m}$; (v) Momentum *p*: p=mV illustrates the price; and (vi) Force *F*: $F=\frac{\mathrm{dp}}{\mathrm{dt}}$ represents the desire or want, where $\frac{\mathrm{dmeasure}}{\mathrm{dt}}$, of measures *q*, *V* and *p* is the first derivative of the measures over time, *t*.

Based on the concept of velocity, since SID refers to the level of demand that exists beyond what would occur in a market where consumers possess complete information, we can write the equations $V=V_{\mathrm{Physician}}-V_{\mathrm{User}}$ or $V=V_{\mathrm{Real}}-V_{\mathrm{Ideal}\;}$ where $V_{\mathrm{Physician}}=V_{\mathrm{Real}}\;$ is the demand induced by the physician, while $V_{\mathrm{User}}=V_{\mathrm{Ideal}\;}$ is the demand that would be induced by a well-informed user. The equations $V=V_{\mathrm{Physician}}-V_{\mathrm{User}}$ or $V=V_{\mathrm{Real}}-V_{\mathrm{Ideal},}\;$ represent the relative velocity or excess demand, where $V_{\mathrm{Physician}}>V_{\mathrm{User}}$ or $V_{\mathrm{Real}}>V_{\mathrm{Ideal}}$. At this point, we should make it clear that in our framework, excess demand refers to a relativistic concept; it does not represent the condition that exists when the demand for a service exceeds its supply at the prevailing price (12). In addition, we should note that demand refers to desire plus ability and willingness to enact that desire (13). Thus, since desire is the motivating force behind consumption (14), based on the second law of Newton:

$$F=\mathrm{SID}=m\frac{\mathrm{dV}}{\mathrm{dt}}+V\frac{\mathrm{dm}}{\mathrm{dt}}-m\frac{\mathrm{dV}*}{\mathrm{dt}}=m\left(\frac{dV_{\mathrm{Physician}}}{\mathrm{dt}}-\frac{dV_{\mathrm{User}}}{\mathrm{dt}}\right)+(V_{\mathrm{Physician}}-V_{\mathrm{User}})\frac{\mathrm{dm}}{\mathrm{dt}}-m\frac{\mathrm{dV}*}{\mathrm{dt}}$$ (1)

Through the analogy between Newton's second law and the law of demand, the desire F=SID, relates to a movement along (first part of the sum), or a rotation of the demand curve (second part of the sum). In addition, a shift in the demand curve corresponds to a fictitious force that arises due to a comparison with an elastic benchmark (third part of the sum); the third part, i.e., $-m\frac{\mathrm{dV}*}{\mathrm{dt}}$ represents a condition in which if the reference market alters its quantity demanded at a rate $\frac{\mathrm{dV}*}{\mathrm{dt}}$, the agent will strive to maintain its original quantity demanded to safeguard its initial demand. To force it to keep up with changing market conditions, it must be provided with an incentive $-m\frac{\mathrm{dV}*}{\mathrm{dt}}$ (11). In addition, since SID is linked to time, i.e., longer treatments than necessary (15), we may argue that changes in the demand over time are more under the physician's control than under the patient's, thus $\frac{dV_{\mathrm{Physician}}}{\mathrm{dt}}>\frac{dV_{\mathrm{User}}}{\mathrm{dt}}$.

At this point, we should note that healthcare users have knowledge regarding physicians' recommendation strategies (16). However, we should highlight that knowledge of recommendation strategies differs from knowledge regarding health, diagnosis and treatment, i.e., asymmetric information.

Notably, Equation 1 concerns the healthcare user, who is subject to two types of demand, one induced by the physician and one induced by himself. The reason we apply the second law of Newton is that even though such types of demand are distinguishable, the demander faces a total demand.

**Figure 1:**
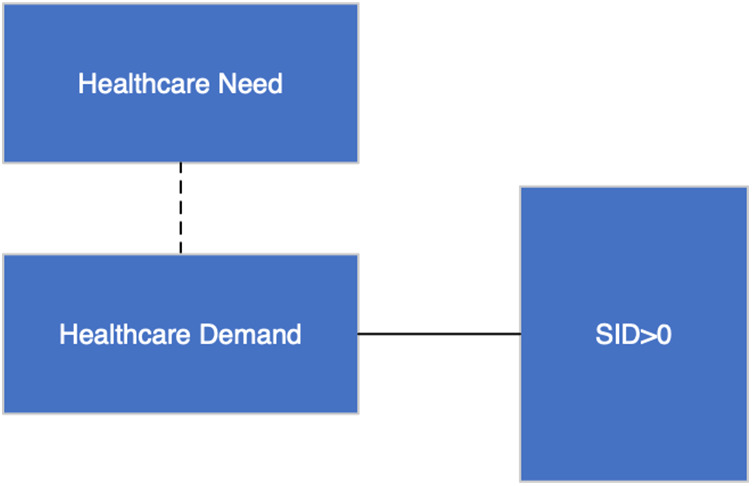
Supplier-induced demand (SID > 0). The dashed line denotes “not linked”, whereas the solid line denotes “are met”.

On the basis of Equation 1, the following cases are valid: (i) SID≅0, when $\frac{dV_{\mathrm{Physician}}}{\mathrm{dt}}≅\frac{dV_{\mathrm{User}}}{\mathrm{dt}}$, $V_{\mathrm{Physician}}≅V_{\mathrm{User}}$, and $m\frac{\mathrm{dV}*}{\mathrm{dt}}=0$, meaning that physicians act almost exclusively according to patients' needs. In addition, $m\frac{\mathrm{dV}*}{\mathrm{dt}}=0$ indicates that the reference market has not changed its quantity demanded; the symbol ≅ was used to be consistent with the definition given the condition $V_{\mathrm{Physician}}>V_{\mathrm{User}}$; (ii) SID>0, when $\frac{dV_{\mathrm{Physician}}}{\mathrm{dt}}>\frac{dV_{\mathrm{User}}}{\mathrm{dt}}$, $V_{\mathrm{Physician}}>V_{\mathrm{User}}$ and $m\frac{\mathrm{dV}*}{\mathrm{dt}}<m\left(\frac{dV_{\mathrm{Physician}}}{\mathrm{dt}}-\frac{dV_{\mathrm{User}}}{\mathrm{dt}}\right)+(V_{\mathrm{Physician}}-V_{\mathrm{User}})\frac{\mathrm{dm}}{\mathrm{dt}}$, meaning that physicians induce demand, even though the incentive is provided to healthcare users; and (iii) SID<0, when $\frac{dV_{\mathrm{Physician}}}{\mathrm{dt}}>\frac{dV_{\mathrm{User}}}{\mathrm{dt}}$, $V_{\mathrm{Physician}}>V_{\mathrm{User}}$ and $m\frac{\mathrm{dV}*}{\mathrm{dt}}>m\left(\frac{dV_{\mathrm{Physician}}}{\mathrm{dt}}-\frac{dV_{\mathrm{User}}}{\mathrm{dt}}\right)+$ $(V_{\mathrm{Physician}}-V_{\mathrm{User}})\frac{\mathrm{dm}}{\mathrm{dt}}$ (Figure 1). The latter implies that SID encompasses care beyond the standard, which may positively impact health, making it both beneficial and necessary (17, 18), thus avoiding subsequent use of healthcare services.

Furthermore, in the case of SID > 0, since $\frac{dV_{\mathrm{Physician}}}{\mathrm{dt}}-\frac{dV_{\mathrm{User}}}{\mathrm{dt}}>0$, the physicians evaluate their needs more highly than those of the users. This confirms that they do not behave as ideal agents but are in line with their personal interests. Thus, the term $m\left(\frac{dV_{\mathrm{Physician}}}{\mathrm{dt}}-\frac{dV_{\mathrm{User}}}{\mathrm{dt}}\right)$ indicates that price alterations induce demand, something that is made more clearer through the term $(V_{\mathrm{Physician}}-V_{\mathrm{User}})\frac{\mathrm{dm}}{\mathrm{dt}}$. That is, SID not only refers to induced overconsumption (19) but also refers to price differentiation (20), although the latter is not the rule. The rationale is that physicians understand that when inelasticity m is zero, SID also becomes zero, which is evident in the model.

Healthcare users, even though they understand the concept of SID, fail to control it. The main reason is physicians' behavior, which is related not only to the health status of the patient but also to the payment scheme, the number of physicians, professional uncertainty, the role and standing of the physician in his profession, the legal regulations governing the medical profession, and the patient's level of information (21). The continuing demand induced by physicians, despite healthcare users' knowledge of their recommendation strategy, increases uncertainty regarding health, diagnosis, and treatment. In this case, both patients' and professionals’ uncertainty is reflected in the varying effects of the economic forces, i.e., SID≅0 or SID>0 or SID<0.

## 3 Discussion

The econophysics approach to SID can directly integrated with the wider literature, through the influence of: (i) agency; (ii) uncertainty; (iii) economic forces; and (iv) incentives, as presented in the previous section.

Based on the traditional approach, under SID, the physician's actions shift the patient's demand curve toward the physician's own benefits. Physicians can influence this shift since they possess more information about the patient's condition and treatment choices than the patient, illustrating a type of market failure called asymmetric information (22). However, the agency relationship between physicians and patients arises not simply because of asymmetrical information but from an asymmetrical ability and willingness to exercise judgement in the face of uncertainty. In this context, the relationship between physicians and patients develops not solely due to asymmetric information but also from a disparity in the capacity and willingness to make decisions when confronted with uncertainty. When considering the uncertainty in medical decision-making and the intricacies of medical judgments, SID emerges as a more credible explanation of the behaviors of patients and physicians compared to the conventional demand and supply model (23). Thus, uncertainty is one of the primary enablers of SID (24). Thus, since our model confirms both the influence of agency and uncertainty on demand inducement, it is in line with the traditional approach.

On the other hand, although it is difficult to interpret the forces promoting SID (25), the influence of such forces is confirmed by Dyckman (26), as cited by McGuire (27), since referring to physicians' capacity to induce demand, he argued that “normal market forces are weak or nonexistent”, indicating that studying SID through the action of economic forces is valid. Moreover, the terms “desire” and “want” in economics indicate the presence of economic forces (11). This is the third pillar indicating that the econophysics approach to SID can be directly integrated with the wider literature. The fourth pillar concerns incentives, setting limits for physicians to follow beneficial treatment schemes.

Regarding policy making against SID, one may first study the multiple factors that contribute to it. These factors pertain to the healthcare system, the insurer, the healthcare provider, and the healthcare user, while their understanding acts as a driving force in health policy making. Monitoring and control, fee-for-service payment systems, medical guidelines and protocols, the nature of the health system, weak health information technology infrastructures, ineffective referral systems, financing techniques, dysfunctional tax systems, and health policies are factors linked to the healthcare system, while the limited involvement of insurance companies, inadequate oversight of insurance firms, lack of advancement in private insurance and supplemental insurance, breaches of insurance regulations and agreements, and insufficient development in evidence-based policy making are factors associated with the insurer. Factors associated with the healthcare provider include the provider's interests, legal repercussions (legal and medicolegal responsibilities), non-medical indications for requests, agency, the employment agreement status of physicians, and the impact of pharmaceutical companies and medical device manufacturers, while cultural concerns and patients' lack of information, low deductibles, and distrust in the nation's health system are among the factors associated with the healthcare user (28).

Based on the information provided, a combination of several policies is necessary, including reforms in reimbursement, quality assurance initiatives (potentially involving monitoring and audits), the creation and sharing of clinical guidelines, increased funding for research on outcomes (encompassing cost-effectiveness, cost-utility, and cost–benefit analyses), sharing information on effectiveness and appropriateness with the public, enhancing methods for gathering informed public preferences regarding healthcare resource distribution, and modifying the physician–patient interaction model and decision-making process (possibly starting in medical schools) to empower patients and support informed decision-making; hence, the three policy goals should be: (i) to decrease or remove ineffective use; (ii) to enhance the effectiveness with which physicians act on behalf of their patients; and (iii) to increase allocative efficiency in utilization, even when (i) and (ii) above are not relevant (16).

The policy implications of the model are related to the fact that it reveals the conditions under which SID becomes positive. Such conditions highlight the need to continuously inform patients about several aspects of SID, such as physicians' behavior, asymmetric information, uncertainty and also patients’ behavior. Improving health literacy and health system literacy could be catalytic in “braking” the mechanism that creates SID. On the other hand, attention should be paid to the incentives provided to decrease the intensity of SID. Although it would be an illusion to expect to completely eliminate asymmetric information or uncertainty, it would not be unreasonable to set a target to change patients' behavior or seek efficient incentives make it easier to approach the ideal demand. Since the factors contributing to SID are known, it is easier to plan how to isolate them. In policy-making, the first and most critical problem is the diagnosis of the problem. But this step has been completed. On the other hand, physicians induce demand because they can do so. Thus, it is not an exaggeration to say that SID exists because the conditions that favor it exist. To solve the problem, we should first focus on patients, secondly on the inefficiencies of the healthcare system, and finally on physicians through mechanisms that could inhibit actions. The aforementioned tasks require the collaboration of several scientific disciplines, such as health policy, health services research, health economics, behavioral economics, and econophysics. Moreover, considering that SID is multidimensional or, in other terms, there is evidence of SID in healthcare amidst a complex interaction of numerous variables (29), system science could also contribute to recognizing the critical role of interactions and boundaries between subsystems to understand the whole. Although it is difficult to distinguish the boundaries of the healthcare system, because it is an open system, its study may provide information regarding its inefficiencies.

The fact that econophysics draws conclusions even if it starts from the adoption of definitions confirms Wylie (1970), who argued that the first step in measurement is the definition (30). Thus, the mathematical formalism of econophysics allows us to reproduce the results through a single equation indicating that there are patterns requiring monitoring. However, the integration of the econophysics model with empirical literature is not possible, as it is the first time supplier-induced demand is studied through Newtonian physics.
